# Molecular imaging predicts trastuzumab‐deruxtecan (T‐DXd) response in head and neck cancer xenograft models

**DOI:** 10.1002/1878-0261.70056

**Published:** 2025-05-28

**Authors:** Abdullah Bin Naveed, Lucas Mani, Muhammad Bilal Mirza, Ashtyn McAdoo, Takahito Kondo, Hidenori Tanaka, Nicole Meeks, Eben Rosenthal, Marisa Hom

**Affiliations:** ^1^ Department of Otolaryngology Vanderbilt University Nashville TN USA; ^2^ Department of Surgery Vanderbilt University Nashville TN USA

**Keywords:** antibody‐drug conjugates, drug delivery, Erb‐b2 receptor tyrosine kinase 2, head and neck squamous cell carcinoma, mice xenograft, optical imaging

## Abstract

Erb‐b2 receptor tyrosine kinase 2 (*ERBB2*; also known as *HER2*) expression is observed in 25–40% of head and neck squamous cell carcinomas (HNSCC), yet there are no anti‐*HER2* therapies under evaluation for HNSCC, as conventional cytostatic anti‐*HER2* antibodies have had limited effectiveness and levels of *HER2* overexpression are lower in HNSCC tumors compared to breast cancer. Trastuzumab‐deruxtecan (T‐DXd; Enhertu) is a *HER2*‐targeting antibody‐drug conjugate (ADC) comprising an anti‐*HER2* monoclonal antibody, a cleavable linker, and a potent topoisomerase I inhibitor payload, and has shown promising results in very low *HER2‐*expressing tumors. We compare the efficacy of T‐DXd, trastuzumab‐emtansine (ADC comprising an anti‐*HER2* antibody and microtubule inhibitor, T‐DM1; Kadcyla) and trastuzumab (Herceptin) therapy in HNSCC with low and absent *HER2* expression *in vitro* and *in vivo*. *In vitro* treatment of a low *HER2*‐expressing human HNSCC cell line (FaDu) with T‐DXd resulted in dose‐dependent cell death (IC50 values of 9856 ng·mL^−1^). T‐DXd treatment of FaDu and UMSCC‐47 (low *HER2*‐expressing cell line) mouse xenografts displayed antitumor activity (*P* = 0.0001 and 0.015 respectively). When comparing T‐DXd to other approved anti‐*HER2* therapies, only FaDu mice treated with T‐DXd showed a reduction in tumor growth (*P* = 0.0012). In UMSCC‐1 cells (absent *HER2* expression), the drug failed to accumulate in tumors and showed no measurable antitumor effect, in contrast to FaDu xenografts, where drug accumulation in the tumor correlated with a therapeutic response. T‐DXd treatment yielded antitumor activity in FaDu and UMSCC‐47 tumors, highlighting the potential for T‐DXd efficacy in low *HER2‐*expressing tumors.

## Graphical abstract

Trastuzumab‐deruxtecan, a HER2‐targeting antibody‐drug conjugate, shows promising antitumor activity in head and neck squamous cell carcinoma with low HER2 expression. *In vitro* and *in vivo* studies demonstrated dose‐dependent cell death and tumor growth reduction in low HER2‐expressing cell lines, which correlated with drug accumulation measured using a fluorescent antibody‐drug conjugate.

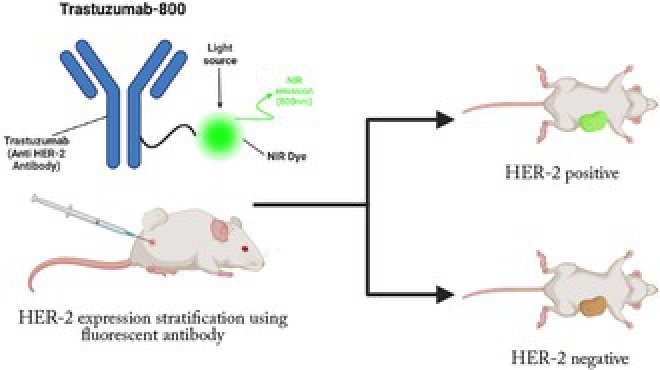

**Abbreviations**
ADC: antibody‐drug conjugate
EPR: enhanced permeability and retention
H&E: hematoxylin and eosin stain
HNSCC: head and neck squamous cell carcinoma
IHC: immunohistochemistry
MFI: mean fluorescent intensity
NIRF: near infrared fluorescence
OTLA: optically labeled antibody
PanCK: pan cytokeratin
SBR: signal‐to‐background ratio
T‐DM1: trastuzumab‐emtansine
T‐DXd: trastuzumab‐deruxtecan

## 1 Introduction

Head and neck squamous cell carcinoma (HNSCC) is the seventh most common cancer in the world, with an estimated 900 000 new cases per year and 400 000 deaths annually [1]. Targeted therapies have not significantly changed the standard of care for this disease, and surgical resection, along with adjuvant chemo or radiotherapy, remain the primary treatment modalities. Antibody‐based treatments in HNSCC, such as cetuximab (epidermal growth factor receptor; *EGFR* monoclonal antibody) or pembrolizumab (*CD274*; also known as *PD‐L1* monoclonal antibody) are approved, but mostly in the recurrent/metastatic disease. Yet, the efficacy of these treatments, when used independently, fails to surpass a 20% response rate [2, 3], underscoring a critical gap in the availability of novel, targeted therapeutic solutions for HNSCC.

Human epidermal growth factor receptor 2 (*HER2*, also known as *ERBB2*), a tyrosine kinase receptor that spans the cell membrane, plays a crucial role in promoting cell growth, differentiation, and survival mechanisms [4]. *HER2*‐directed therapy is approved for cancers, such as unresectable or metastatic breast cancer, advanced gastric and gastroesophageal junction cancers, as well as certain colorectal cancers and *HER2*‐mutant lung cancers. Using conventional cytostatic anti‐*HER2* therapies in HNSCC has failed in the past, which is not surprising given the low *HER2* expression (IHC 1+) in HNSCC tumors. However, approximately 40% of HNSCC patients do show some *HER2* expression [5, 6, 7], which means finding an effective anti‐*HER2* therapy for HNSCC treatment might still be worthwhile.

**Fig. 1:**
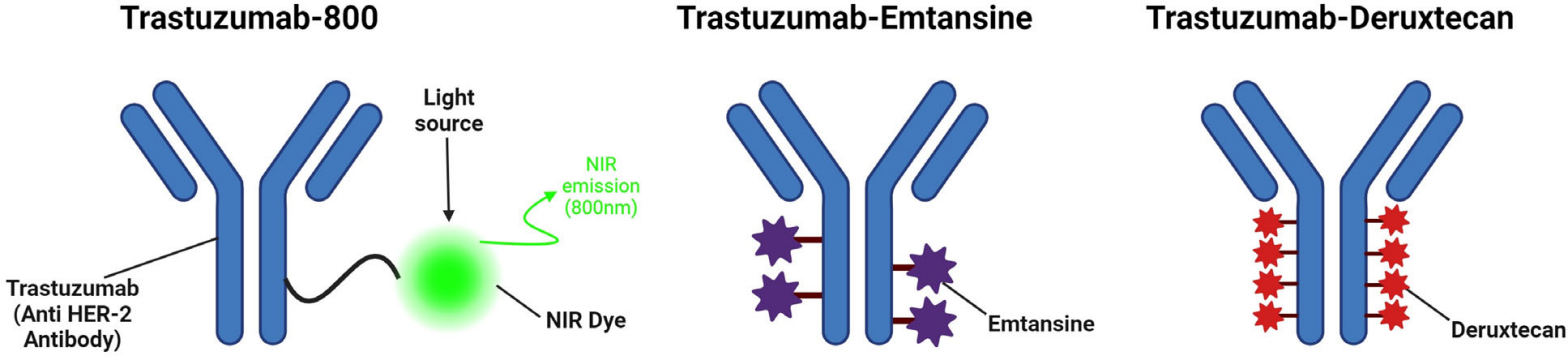
Structure of different *HER2* therapies. Trastuzumab‐800 consists of an IRDye800 fluorophore conjugated to the *HER2* targeting antibody Trastuzumab. Trastuzumab‐Emtansine (T‐DM1) consists of a microtubule inhibitor (emtansine) that binds to tubulin and is linked to Trastuzumab via a non‐reducible thioether linker. Trastuzumab‐Deruxtecan (T‐DXd) consists of a topoisomerase I inhibitor (deruxtecan) that is linked to Trastuzumab via a tetrapeptide linker that can be cleaved selectively by enzymes. NIR, near infrared.

Monoclonal antibodies like trastuzumab are cytostatic and usually require an abundance of the receptor to be present on the tumor to produce an effect. Antibody‐drug conjugates (ADCs) overcome this problem by linking a toxic payload to the antibody, which allows treatment efficacy in tumors with minimal expression of the receptor that is bound by the antibody. Trastuzumab‐Deruxtecan (T‐DXd, Enhertu), a *HER2* antibody linked to a topoisomerase inhibitor by means of a cleavable linker (Fig. 1) takes use of this principle, and unlike conventional anti‐*HER2* therapies, T‐DXd has shown improved patient outcomes compared to physicians' therapy of choice in patients with metastatic breast or gastric cancer with low immunohistochemical (IHC) expression of *HER2* [8, 9]. The FDA's approval of T‐DXd as the first ADC for breast cancer with low *HER2* expression opens promising avenues for treating other cancers characterized by low *HER2* levels. To our knowledge, *HER2*‐targeting ADCs have not been tested in HNSCC. Given T‐DXd's approval for other solid organ cancers, testing it in HNSCC may face fewer regulatory barriers. Given that *HER2* and *EGFR* pathways have significant overlap, targeting both *HER2* and *EGFR* simultaneously could amplify antitumor effects and reduce the likelihood of resistance development, which is a common issue with *EGFR*‐based therapies in HNSCC.

Here, we present data demonstrating the efficacy of anti‐*HER2* targeting ADCs in both *in vitro* and *in vivo* settings and quantify *in vivo* and *ex vivo* drug distribution. In this study, we demonstrate the value of T‐DXd for treating HNSCC xenografts in low *HER2*‐expressing HNSCC.

## 2 Materials and methods

### 2.1 Antibody‐drug conjugates (ADC), antibodies and proteins

Trastuzumab (Herceptin), procured from the Vanderbilt pharmacy, underwent conjugation to the fluorophore utilizing Zeba™ Spin Desalting Columns (catalog #89891; ThermoFisher, Waltham, MA, USA) and a Li‐Cor IRDye® 800CW Protein Labelling Kit (catalog #929‐70020; Li‐Cor, Lincoln, NE, USA). The process has been previously published [10] but briefly, the process involved diluting concentrated trastuzumab in sterile PBS to a concentration of 1 mg·mL^−1^, followed by pH adjustment to 8.5. Subsequently, a Desalting Spin Column was prepared according to the manufacturer's guidelines to eliminate any preservatives from the stock trastuzumab. The purified sample was then incubated at room temperature for 4 h with 3 m equivalents of IRDye800CW, shielded from light. Excess dye was eliminated using a Zeba™ Spin Desalting Column, resulting in a drug concentration of 1.0 mg·mL^−1^ and a dye/protein ratio of 1.22. The resultant product was stored at 4 °C, protected from light. Additionally, both trastuzumab‐emtansine (ADC compromising of anti‐*HER2* antibody, a non‐cleavable linker and microtubule inhibitor, T‐DM1, Kadcyla) and trastuzumab‐Deruxtecan (T‐DXd, Enhertu) were procured commercially from the Vanderbilt pharmacy and stored at 4 °C in accordance with manufacturer recommendations.

### 2.2 Cell culture

The human tumor cell lines FaDu (RRID: CVCL_SA75, established from a hypopharyngeal tumor, purchased from ATCC catalog #HTB‐43™, Manassas, VA, USA), UMSCC‐1 (RRID: CVCL_7707, established from a floor of mouth tumor, purchased from Millipore Sigma, catalog #SCC070, Burlington, MA, USA), UMSCC‐47 (RRID: CVCL_7759, established from a lateral tongue tumor, purchased from Millipore Sigma, catalog #SCC071) and SKBR‐3 (RRID: CVCL_0033, established from a breast tumor, purchased from ATCC catalog #HTB‐30™). UMSCC‐47 and UMSCC‐1 cells were cultured in Dulbecco's modified Eagle medium (DMEM, catalog #11965092; Gibco/ThermoFisher Scientific, Waltham, MA, USA) supplemented with 1% penicillin–streptomycin (catalog #15‐070‐063; Gibco/ThermoFisher Scientific), 1% non‐essential amino acids (NEAAs, catalog #11140050; Gibco/ThermoFisher Scientific), and 10% fetal bovine serum (FBS) (catalog #S11150‐NOV; Bio‐Techne/Fischer Scientific). FaDu cells were maintained in DMEM/Nutrient Mixture F‐12 (DMEM/F‐12, catalog #11320033; Gibco/ThermoFisher Scientific) supplemented with 1% penicillin–streptomycin (catalog #15140122; Gibco/ThermoFisher Scientific), 400 ng·mL^−1^ hydrocortisone (catalog #NC1062339; Stemcell Technologies/Fisher Scientific), and 10% FBS (Bio‐Techne/Fischer Scientific). SKBR‐3 cells were maintained in McCoy's 5a Medium Modified (catalog #30‐2007; ATCC) supplemented with 10% FBS. All cells were cultured at 37 °C and 5% CO_2_. Before beginning the experiments, the cell lines were authenticated by routine short tandem repeat analysis provided by ATCC and assessed for Mycoplasma contamination by using Hoechst‐33258 fluorescent dye.

### 2.3 Flow cytometry

For analysis of *HER2* expression on cell lines, cells were first incubated with live/dead fixable aqua dead cell stain (L34957; ThermoFisher Scientific) in PBS for 15 min at 4 °C. Subsequently, cells were incubated with a *HER2* monoclonal antibody (324410; Biolegend, San Diego, CA, USA) for 15 min at 4 °C. Samples were acquired on a BD FACSCelsta flow cytometer with 10 000–40 000 cells analyzed per sample. The SKBR‐3 cell line (*HER2*‐positive breast cancer cell line) was used as a positive control for the antibody. Stained samples mean fluorescent intensity (MFI) were compared to their non‐stained controls MFI to determine *HER2* expression. Results were tabulated using the flowjo software, (Ashland, OR, USA).

### 2.4 Immunohistochemical analysis

Immunohistochemistry was performed on formalin‐fixed paraffin‐embedded tumor sections (5 μm thick). Slides were placed on the Leica IHC Bond stainer. All steps besides dehydration, clearing, and cover slipping are performed on the Leica Bond. Slides were deparaffinized, and heat‐mediated antigen retrieval was done with their Epitope Retrieval 1 proprietary antigen retrieval solution (AR9961; Leica, Teaneck, NJ, USA) for 20 min. The Bond Polymer Refine detection system was used for visualization. Slides were then dehydrated, cleared, and cover slipped. Primary antibodies were: *HER2* human samples (prediluted, PA0571; Leica BioSystems), *HER2* xenografts (1 : 500 dilution, 4290; Cell Signaling, Danvers, MA, USA) and γH2AX (1 : 300 dilution, 05‐636; Millipore). Slides were scored for *HER2* by a board‐certified pathologist and categorized as either 0 (no expression), 1+ (low expression), 2+ (moderate expression) or 3+ (strong/high expression) using the College of American Pathologists recommended interpretation criteria (0 = no staining or membrane staining in less than 10% of tumor cells, 1+ = faint or barely perceptible membrane staining in more than 10% of the cells, with only part of their membrane stained, 2+ = weak to moderate complete membrane staining observed in more than 10% of tumor cells, 3+ = strong complete membrane staining in more than 10% of tumor cells). γH2AX staining was used to monitor treatment response in tumors because it allows for the direct visualization and quantification of DNA double‐strand breaks, which are indicative of DNA damage induced by the payload of the therapeutic agents i.e., T‐DXd and T‐DM1. This approach provides a sensitive and early marker of the effectiveness of treatments such as chemotherapy and radiation therapy in targeting and damaging the DNA within tumor cells, enabling a direct assessment of their impact on cancer cells at the molecular level.

### 2.5 *In vivo* efficacy of T‐DXd

All experiments used female nude mice (NU/NU, Crl:NU‐Foxn1nu) purchased from Charles River (catalog #24102241), aged 4–6 weeks, and quarantined for 1 week before experiments began. Mice were housed under Institutional Animal Care and Use Committee guidelines and all mice experiments met the requirements of Vanderbilt's Animal Care and Use Program. The experiments were carried out and approved under Protocol# M2100079‐00. All models were established by subcutaneous inoculation into the left flank of the mice. FaDu, UMSCC‐1, and UMSCC‐47 models were established by injecting 1 × 10^7^, 2 × 10,^7^ and 1 × 10^7^ cells in a 1 : 1 mixture of sterile DPBS (catalog #14‐080‐055; ThermoFisher Scientific) and Matrigel (catalog #CB‐40234A; ThermoFisher Scientific). For treatment efficacy experiments, tumor volumes were allowed to reach approximately 100–200 mm^3^ before the tumor‐bearing mice were randomized into treatment and control groups based on the tumor volume, and dosing was initiated (Day 0). In the initial experiment, UMSCC‐47 and FaDu xenografts were treated with either doses of 2 or 4 mg·kg^−1^ of T‐DXd to establish the efficacy of the drug (*n* = 2–3). In the next experiment, FaDu xenografts only were treated with either trastuzumab‐Emtansine, trastuzumab‐Deruxtecan, trastuzumab, or control saline at a dose of 10 mg·kg^−1^ in each group (*n* = 3–5). The tumor volume was defined as 1/2 × length × width^2^. All mice were sacrificed after 21 days or if they reached humane endpoints (tumor ulceration or tumor volume > 1500–2000 mm^3^). Unless specifically mentioned, all mice in their cohort received one dose of the drug (i.e., T‐DXd, T‐DM, Normal Saline etc.) on day 0.

### 2.6 Fluorescent imaging of trastuzumab‐IRDye800 distribution

After being injected with trastuzumab‐IRDye800, a cohort of mice with tumors from the FaDu and UMSCC‐1 cell lines were sacrificed after 72 h (*n* = 2–3). Using a PEARL Trilogy small animal imaging system (LI‐COR Biosciences), the mice were imaged (800 nm; WLR) prior to injection, immediately post‐injection, and 24‐, 48‐, and 72‐h post‐injection. At 72 h, the mice were sacrificed, and organs were excised and imaged. Signal‐to‐background ratios of each organ were measured by normalizing the mean fluorescent intensity to muscle tissue. For assessing the microscopic distribution of the IRDye800 optical agent, FFPE fixed tissue slides were imaged with a VS‐200 slide scanner (Olympus, Center Valley, PA, USA) with the following parameters to image: excitation (760 nm), emission (830 nm) and exposure time (1.1 s). Images were thresholded to a binary positive or negative signal on a pixel‐by‐pixel basis, using an unstained slide of the same tumor type as a negative control. The cutoff for determining binary signals was set based on the average pixel intensity of the unstained tumor slide, imaged under the same parameters.

### 2.7 Cytotoxicity assay

Cells were seeded in 96‐well plates (black walled, clear bottom, catalog #164588; ThermoFisher Scientific) at a density of 2 × 10^2^ cells/well and cultured overnight in complete growth media. Following approximately 12 h of incubation, the cells were 70–80% confluent. The media was aspirated, and fresh media was added containing titrations of T‐DXd, T‐DM1, and trastuzumab (0–100,000 ng·mL^−1^). The cells were incubated at 37 °C for 72 h in a humidified, 5% CO_2_ atmosphere. Cell viability was determined using a CellTiter96® Aqueous One Solution Cell Proliferation Assay (catalog #G3582; ThermoFisher Scientific). Twenty microliter of CellTiter96^®^ Aqueous One was added to each well, and the plate was allowed to incubate at 37 °C for 3 h. The relative absorbances at 490 nm were used to determine cell viability. Absorbance from no drug control was used as a reference for 100% cell viability. A detailed version of this method has also been published by our lab [10].

### 2.8 Statistical analysis

Data is expressed as mean ± standard error of the mean (SEM). All comparisons between two groups were performed using unpaired *t*‐tests (two‐tailed). Tumor growth rate inhibition analysis between groups was performed using a type II ANOVA test using the TumGrowth package. IC50 values were determined by nonlinear regression analysis of concentration response curves. All data was analyzed using (graphpad prism 10.0, Boston, MA, USA) unless otherwise stated. Significance was defined at *P* < 0.05.

## 3 Results

### 3.1 *HER2* expression levels in HNSCC cell lines and tumors

**Fig. 2:**
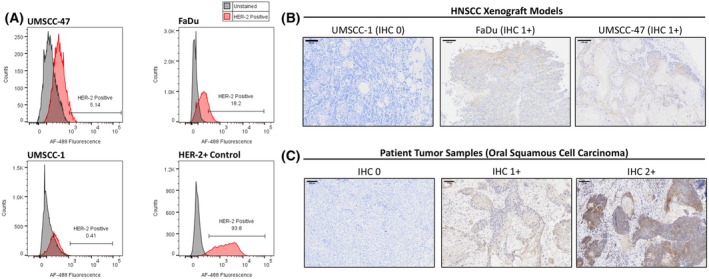
Analysis of *HER2* expression across head and neck cell lines and human tumor samples. (A) Flow cytometry analysis of *HER2* expression (red) versus unstained control (gray) in 10 000–40 000 cells for each cell line. FaDu and UMSCC‐47 cells expressed low *HER2* expression, whereas UMSCC‐1 cells had negligible *HER2* expression. (B) UMSCC‐1, FaDu, and UMSCC‐47 *HER2* IHC stain in their xenograft models. UMSCC‐1 cell line exhibited no *HER2* expression, whereas other cell lines showed low *HER2* expression (IHC 1+) (*n* = 3 each), Scale bar = 100 μm. (C) *HER2* expression in HNSCC human samples showed varying *HER2* expression ranging from no (0) expression to moderate (2+) expression (*n* = 40), Scale bar = 100 μm. HNSCC, head and neck squamous cell carcinoma; IHC, immunohistochemistry.

Three candidate HNSCC cell lines (UMSCC‐47, FaDu, UMSCC‐1) were evaluated by flow cytometry as well as a reference standard, the breast cancer SKBR‐3 cell line. The fluorescence intensity of all stained cells was compared with that of non‐stained counterparts to establish a cutoff for *HER2* receptor expression. In the SKBR‐3‐positive control, 93.8% of the cells expressed *HER2*. The expression rates for UMSCC‐47, FaDu, and UMSCC‐1 were 5.14%, 18.2%, and 0.41%, respectively (Fig. 2A). To determine if the *HER2* expression *in vitro* and *in vivo* was similar, we evaluated *HER2* expression in tumor xenografts, which showed absent *HER2* expression in UMSCC‐1, whereas FaDu and UMSCC‐47 both had low *HER2* expression. These were scored as 0, 1+, and 1+, respectively, by a pathologist based on the scoring criteria established for breast cancer (Fig. 2B). These results suggest that a 1+ scoring on IHC corresponds to about 5–20% of tumor cells expressing *HER2* on flow cytometry, whereas a score of 0 on IHC would equate to less than 1% of cells expressing *HER2*.

For comparison to human tumors, we evaluated *HER2* protein expression in 40 patients (Table S1) with oral cavity tumors. *HER2* protein immunochemistry (IHC) reactivity was scored (0, 1+, 2+ and 3+) by a board‐certified head and neck pathologist based on breast staging guidelines (Fig. 2C). We found that 20 (50%) of the 40 patients expressed no *HER2* protein (Score = 0) and 50% of the patients expressed some *HER2* as +1 or greater. Tissues from four patients also contained normal mucosa which had no *HER2* immunoreactivity. These results suggest that almost 50% of HNSCC patients express low (1+) to moderate (2+) levels of *HER2* and could potentially benefit from *HER2*‐based therapies and imaging agents.

### 3.2 *In vivo* measurements of *HER2*‐targeted therapy in xenograft model

**Fig. 3:**
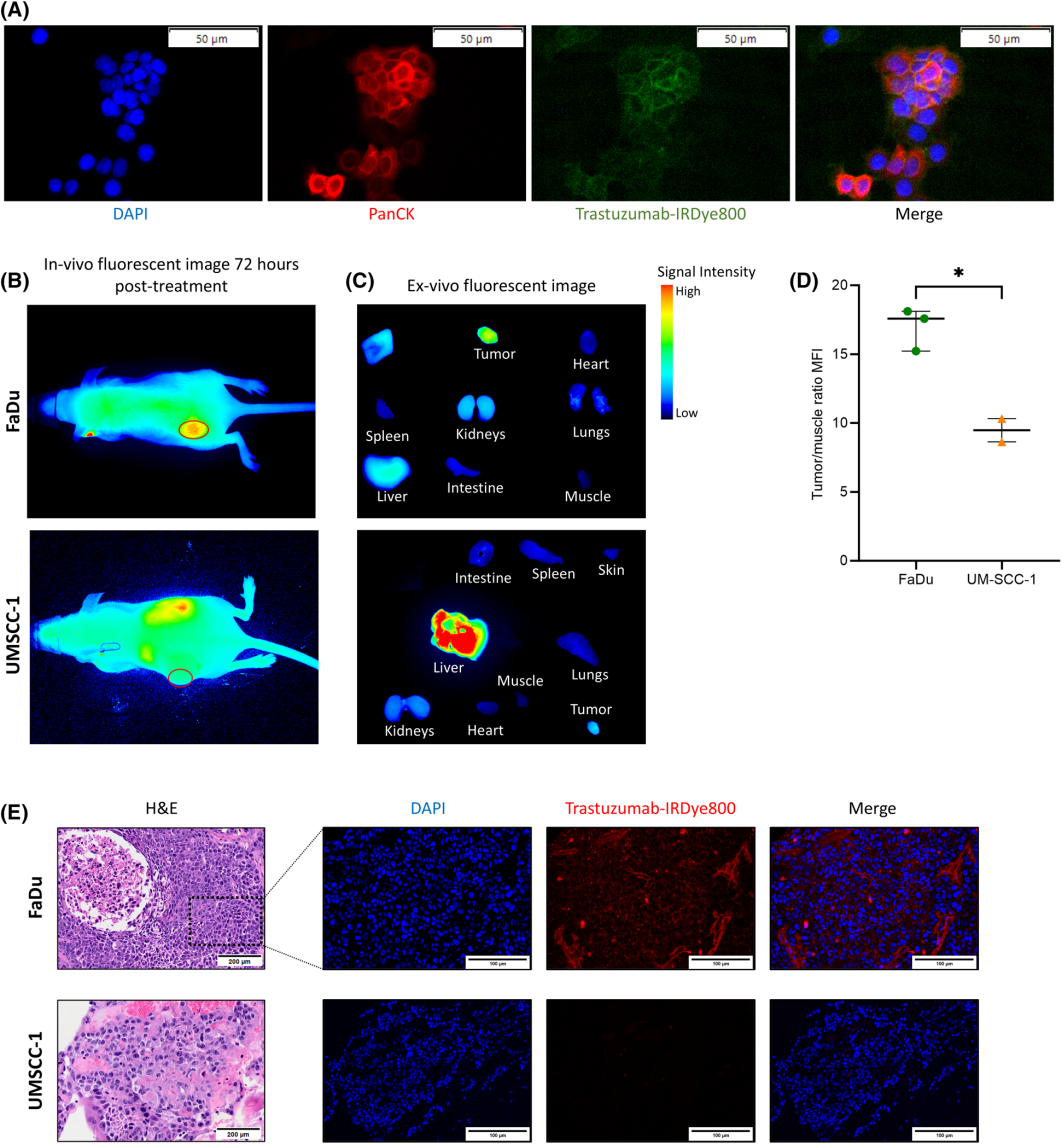
*In vivo* and *ex vivo* imaging of Trastuzumab‐IRDye800 to identify *HER2* negative vs. positive xenografts. (A) High magnification view of FaDu cells incubated with Trastuzumab‐IRDye800 showing positive binding at 24 h post incubation (*n* = 3), Scale bar = 50 μm. (B) *In vivo* NIRF images of Trastuzumab‐IRDye800 in FaDu (top) and UMSCC‐1 (bottom) xenografts. Drug shows localization in FaDu xenograft but not in UMSCC‐1 xenograft at 72 h. Fluorescent signal seen on flank opposite to tumor at 72 h represents liver. Red circle represents where tumor xenograft cells were injected. (C) *Ex vivo* NIRF images of resected organs in FaDu and UMSCC‐1 xenografts at 72 h post Trastuzumab‐IRDye800 injection. (D) Drug uptake in tumors was quantified as MFIs (±SD) normalized to that of muscle, *P < 0.05 between tumors from FaDu and UMSCC‐1 xenograft mice. *P*‐values derived from an unpaired *T*‐test. (E) Representative H&E (Scale bar = 200 μm) and NIRF (Scale bar = 100 μm) images for Trastuzumab‐IRDye800 uptake co‐related with *ex vivo* findings from FaDu xenografts in 5 μm sections (Top). Representative images from UMSCC‐1 xenograft showed no fluorescent signal for Trastuzumab‐IRDye800 (bottom). (B–E) FaDu *n* = 3, UMSCC‐1 *n* = 2; H&E, hematoxylin and eosin stain; MFI, mean fluorescent intensity; NIRF, near infrared fluorescence; PanCK, pan cytokeratin.

To determine if the concentration of drug in the tumor correlated with response, we measured anti‐*HER2* drug delivery to in a xenograft mouse model. To this end, we assessed the parent targeting antibody trastuzumab, conjugated to a fluorophore to track *in vivo* binding; trastuzumab‐IRDye800 was utilized as an imaging probe to identify if drug uptake in tumor co‐related to *HER2* expression that was identified by flow cytometry and IHC. *In vitro* incubation of FaDu cells with trastuzumab‐IRDye800 showed binding of drug to surface which validated binding of the bioconjugate and the overexpression of *HER2* in FaDu cells (Fig. 3A). *In vivo* near infrared fluorescence imaging was then utilized to assess trastuzumab‐IRDye800 uptake in xenograft mice models. Since our previous experiments demonstrated that UMSCC‐1 cell line had no *HER2* expression (IHC 0+, Flow = 0.41%) whereas FaDu cell line had low *HER2* expression (IHC 1+, Flow = 18.2%), we evaluated these two cell lines to demonstrate the effectiveness of our optically labeled antibody (OTLA) trastuzumab‐IRDye800 to show a differential pattern of drug uptake between *HER2*‐negative vs *HER2*‐positive tumors. UMSCC‐1 xenografts had no drug localization even after 72 h post injection. While UMSCC‐1 xenografts exhibited negligible drug signals *in vivo*, FaDu xenografts displayed notable accumulation, suggesting that even with low *HER2* expression, the drug effectively localizes to tumor (Fig. 3B). Seventy‐two hours post‐injection was determined as an optimal imaging point as it provided the highest signal to background ratio (SBR) in FaDu xenografts between 24 h (SBR = 1.29 ± 0.40), 48 h (SBR = 2.41 ± 0.47) and 72 h (SBR = 7.41 ± 3.28) (Fig. S1A,B). *Ex vivo* fluorescent analysis further corroborated our findings, demonstrating significantly higher drug uptake in FaDu tumors compared to UMSCC‐1, with a mean difference in signal intensity of 7.5 ± 1.3 (a.u.), corresponding to a 1.79‐fold increase of drug accumulation in FaDu xenografts (Fig. 3C,D). This data was consistent with flow cytometry and IHC analysis (UMSCC‐1 is *HER2* negative and FaDu has low expression). Additionally, we observed greater signal intensity in UMSCC‐1 livers compared to FaDu livers at the same time point, suggesting rapid clearance of unbound drug from UMSCC‐1 livers (Fig. 3C and Fig. S1C).

To confirm drug accumulation was specific to *HER2*, we obtained serially sectioned slides from the mouse xenografts for both UMSCC‐1 and FaDu. On NIRF imaging, fluorescence intensity comparisons between FaDu and UMSCC‐1 xenograft sections determined that FaDu had a higher fluorescence intensity compared to the UMSCC‐1 xenografts. After thresholding images for background autofluorescence signal, there was negligible signal intensity in the UMSCC‐1 xenograft, whereas positive signal could be seen in the FaDu xenograft. Microscopic images of the slides depicted that at a cellular level, the signal of injected drug was mainly localized to the cell membrane (Fig. 3E).

### 3.3 Antitumor activity of T‐DXd *in vitro* and *in vivo*

**Fig. 4:**
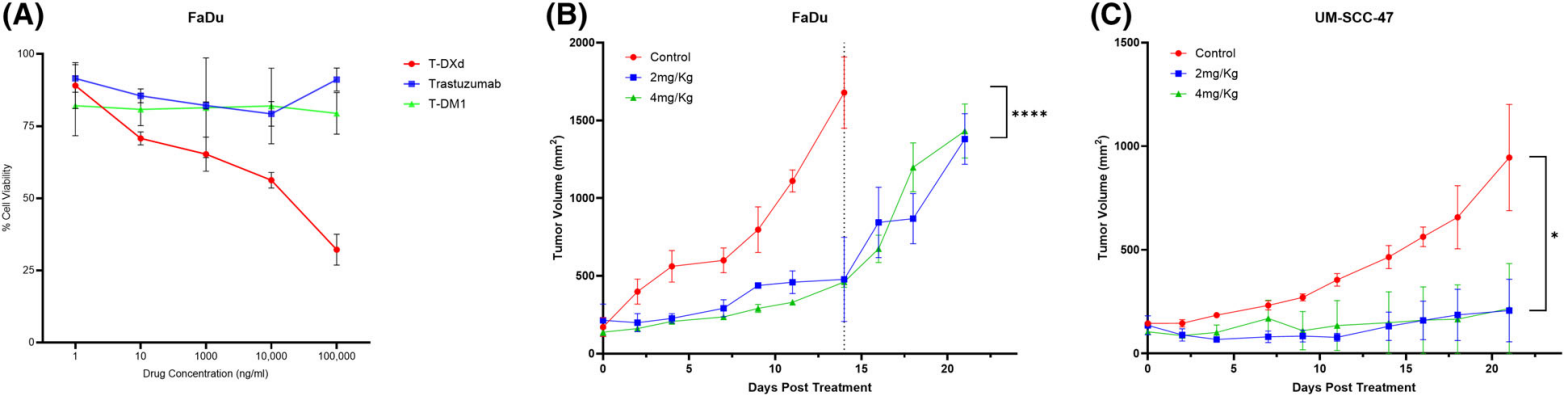
*In vitro* and *in vivo* efficacy of T‐DXd. (A) *In vitro* cell growth inhibitory activity in the FaDu cell line. The cells were treated with T‐DXd, Trastuzumab, and T‐DM1 for 4 days. Each point represents the mean and SD (*n* = 3). (B) Antitumor efficacy of T‐DXd in the FaDu xenograft model. The tumor‐bearing mice were allowed to grow till 150 mm^3^ before being administered one dose of T‐DXd and saline intraperitoneally on day 0. Each point represents the mean tumor volume and SEM, *P*‐values derived from a type II ANOVA test between all treatment cohorts, ****P < 0.0001. The dotted line on the *x*‐axis at day 14 represents the day that mice were sacrificed in the control group due to reaching humane end points. (C) Antitumor efficacy of T‐DXd in the UMSCC‐47 xenograft model. *P*‐values derived from a type II ANOVA test between all treatment cohorts, *P < 0.05. (B, C) Control *n* = 3, Treatment arms *n* = 2.

The inhibitory activity of T‐DXd against cell cancer growth *in vitro* was compared to two other approved anti‐*HER2* agents, trastuzumab (Herceptin) and T‐DM1 (Kadcyla) in the FaDu cell line since it exhibited the highest *HER2* expression in the HNSCC cell lines we tested (IHC 1+, 18.2% via flow and *in vivo* SBR = 7.41 ± 3.28 using optically labeled trastuzumab). The toxic payload of T‐DXd is deruxtecan – a topoisomerase I inhibitor. As such, the cytotoxicity of the drug relies on endocytic internalization and subsequent cleaving of deruxtecan from the antibody, allowing the payload to interact with topoisomerase I. FaDu cells *in vitro* were treated with doses similar to those used in previous studies investigating the toxicity of T‐DXd, ranging from 0 to 100 000 ng·mL^−1^ and incubated for 72 h before cell viability was assessed. Remarkable inhibitory activity to cell growth was observed for T‐DXd against *HER2*‐positive FaDu with the IC_50_ value of 9856 ng·mL^−1^, whereas no such inhibition was seen with trastuzumab or T‐DM1 with the IC_50_ values being > 100 000 ng·mL^−1^ (Fig. 4A). These IC_50_ values show that T‐DXd demonstrates cytotoxic activity in *in vitro* models with low *HER2* expression where other anti‐*HER2* therapies fail.

The *in vivo* antitumor activity of T‐DXd was evaluated in the low *HER2* expressing FaDu and UMSCC‐47 xenograft model to ensure that T‐DXd would work on multiple low *HER2* expressing HNSCC cell lines. UMSCC‐1 was not treated with T‐DXd since we found no *HER2* expression based on our results. T‐DXd induced tumor growth inhibition and tumor regression with a single dose of at least 2 mg·kg^−1^, without inducing any abnormalities in the general condition or body weight of the mice in all the cohorts. In the FaDu xenograft model, the administration of T‐DXd yielded a significant decrease in tumor growth progression (*P* < 0.0001) when compared to the control by day 14. The mean tumor volume in the control group at day 14 was 1679 ± 324 mm^3^ compared to 478 ± 383 mm^3^ and 461 ± 48 mm^3^ in the 2 and 4 mg·kg^−1^ treatment group. We found no difference in tumor growth inhibition between a dose of 2 or 4 mg·kg^−1^, suggesting maximal efficacy of the drug is achieved at lower doses in low *HER2* expressing tumors (Fig. 4B). All mice in the UMSCC‐47 cohort likewise showed a decrease in tumor growth that was statistically significant (*P* = 0.015). Interestingly, one of the tumors in the UMSCC‐47 cohort that was treated with 2 mg·kg^−1^ of drug had complete tumor regression suggesting that the response to the drug has great variability (Fig. 4C). Comparative analysis was done at day 14 rather than day 21 as most mice in the control group reached human end points around day 14, making comparison of T‐DXd treated mice with control mice at day 21 impossible.

**Fig. 5:**
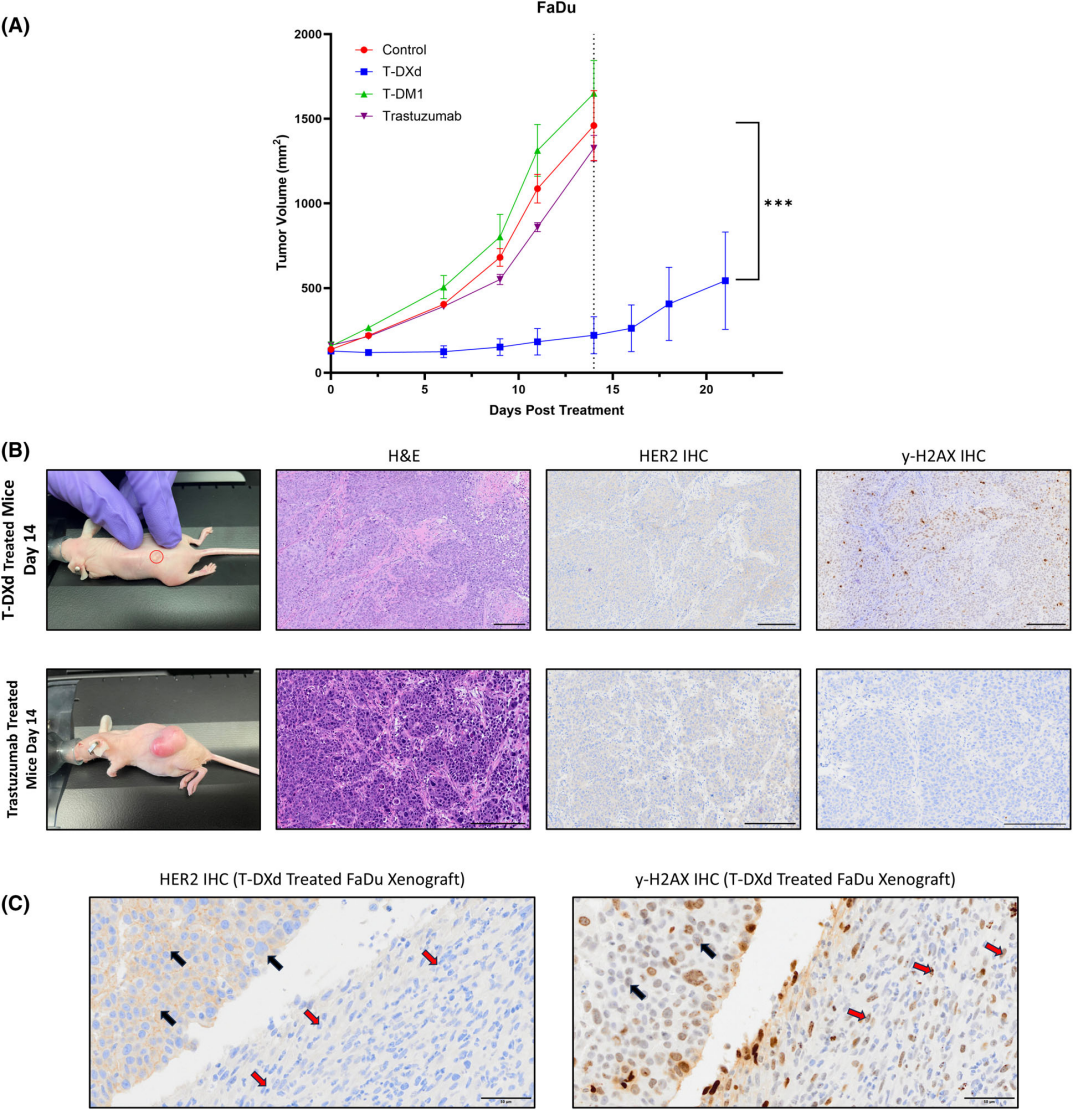
Antitumor activity of T‐DXd compared with T‐DM1 and Trastuzumab in tumors with low *HER2* level. (A) Antitumor activity of T‐DXd compared with T‐DM1 and Trastuzumab in FaDu xenografts. Each point represents the mean and SEM; *P*‐values derived from a type II ANOVA test between all treatment cohorts, and the dotted line on the *x*‐axis at day 14 represents the day that mice were sacrificed in all treatment groups other than T‐DXd due to reaching humane end points. Each mouse was given one dose of 10 mg·kg^−1^ of their treatment cohort on day 0. (B) H&E, *HER2*, and y‐H2AX IHC staining of 5 μm sections of FaDu xenografts treated with either T‐DXd (top) or Trastuzumab (bottom) at day 14 (Scale bar = 200 μm). (C) Representative sections showing *HER2* expression vs y‐H2AX staining in FaDu tumors. Black arrows on the image on the left represent HER2‐positive cells, whereas red arrows show *HER2* negative cells. Black arrows on the image on the right show *HER2*‐positive cells that are also positive for y‐H2AX, whereas red arrows show *HER2* negative cells that are positive for y‐H2AX staining. Scale bar = 50 μm. (A–C) Control and T‐DXd *n* = 5, T‐DM1 *n* = 4, Trastuzumab *n* = 3. H&E, hematoxylin and eosin stain; IHC, immunohistochemistry.

We next evaluated the efficacy of T‐DXd compared to Trastuzumab and T‐DM1 in a FaDu xenograft model. T‐DM1 and Trastuzumab are approved for *HER2*‐positive breast cancers that express either 2+ or 3+ staining on IHC, whereas T‐DXd is approved for *HER2* breast cancers that express 1+, 2+, or 3+ *HER2* expression on IHC. While our *HER2* staging of HNSCC cell lines demonstrated low *HER2* expression (4–18% of cells, 1+ IHC), we wanted to compare all FDA‐approved *HER2* antibodies. T‐DXd demonstrated remarkable efficacy in the FaDu cell line when compared to all other therapies and control (*P* = 0.0012). By day 14, the mean tumor volume observed in the T‐DXd group was 221 ± 246 mm^3^. When compared to this, the control group exhibited a mean tumor volume that was 6.61‐fold greater (1460 ± 413 mm^3^), the T‐DM1 group displayed a 7.47‐fold increase (1650 ± 336 mm^3^), and the trastuzumab group showed a 6.00‐fold rise (1327 ± 131 mm^3^). Remarkably, 2 out of 5 xenografts had complete regression in the T‐DXd cohort, suggesting that even with low *HER2* levels, payload delivery is sufficient to induce cytotoxicity. Neither T‐DM1 nor trastuzumab had a statistically significant reduction in tumor growth when compared to the control (Fig. 5A).

To correlate T‐DXd toxicity in the tumors, we co‐localized the expression of γH2AX and *HER2*. Positive γH2AX staining was seen for T‐DXd treated mice, which did not correlate with *HER2* expression. This suggests effective diffusion of the toxic payload across the cells and targets non‐*HER2* expressing cells. For xenografts treated with Trastuzumab or T‐DM1, γH2AX staining was negative, suggesting there was no downstream effect of either treatment. The ability of the T‐DXd toxic payload to diffuse into neighboring cells regardless of *HER2* status is one hypothesis that explains why T‐DXd works in low *HER2* expressing tumors where T‐DM1 and Trastuzumab have failed (Fig. 5B,C).

## 4 Discussion

Although Trastuzumab, both as a monotherapy and combined with other agents, has not proven effective in HNSCC, no trials have yet explored the use of anti‐*HER2* ADCs, such as T‐DM1 or T‐DXd, in this context. Our data highlight two key findings: first, the therapeutic efficacy of T‐DXd in preclinical xenograft models, and second, that low *HER2* expression levels are sufficient for T‐DXd to be effective, whereas Trastuzumab and T‐DM1 may not yield similar results.

Current data from clinical trials conducted in breast cancer patients suggests that T‐DM1 would be unlikely to be effective in low *HER2* expressing cancers such as HNSCC [11]. This contrasts with studies done on T‐DXd, with results from the DESTINY and DAISY [8, 9, 12] trial demonstrating the efficacy of T‐DXd in both breast and gastric cancers even with low (IHC 1+) *HER2* expression. Surprisingly, there is also some evidence of its utility in *HER2*‐negative patients [12]. To assess T‐DXd's effectiveness in HNSCC, we performed a dose escalation study (2–4 mg·kg^−1^). Increasing the dose came without significant benefit, suggesting that lower doses are sufficient to saturate low *HER2* expressing tumors. Although there was a dose‐dependent effect in preclinical studies that utilized a cell line that had high (IHC 3+) *HER2* expression, there are still some questions that need to be answered with regards to whether high *HER2* expressing tumors would require more drug compared to low *HER2* expressing tumors [13]. Extending our experiment to evaluate all FDA‐approved *HER2* therapies in a FaDu xenograft model, we observed a favorable response only in mice treated with T‐DXd. Microscopic analysis of post‐treatment xenografts revealed high expression of γH2AX in only the T‐DXd cohort, suggesting that T‐DXd was able to exert a therapeutic molecular effect on the tumor, which was consistent with our data that showed the lack of efficacy of trastuzumab and T‐DM1 in HNSCC tumor regression. These findings highlight the potential of T‐DXd in treating HNSCC patients and present an exciting new avenue to be explored for targeted therapy in HNSCC.

In this study, we also demonstrated the utility of using a NIRF labeled *HER2* antibody (Trastuzumab‐IRDye800) by comparing two HNSCC cell lines, FaDu and UMSCC‐1. *In vivo* imaging of the fluorescent conjugate depicted clear tumor localization in low *HER2*‐expressing FaDu xenografts at 72 h post‐injection, while no tumor‐specific fluorescence was seen in mice with UMSCC‐1 xenografts which lack *HER2* expression. The small accumulation of drug in UMSCC‐1 xenografts *in vivo* could be attributed to enhanced permeability and retention (EPR) effect [14, 15] which has been well studied with IgG based antibodies and ICG. Histologic evidence of drug accumulation was clearly seen in the FaDu tumors suggesting that even low *HER2* expression is sufficient for drug accumulation and efficacy. These findings present promising support for the use of Trastuzumab‐IRDye800 in differentiating between *HER2*‐negative and low *HER2*‐expressing tumors [16, 17, 18]. This distinction is crucial in the context of HNSCC, where patients frequently exhibit low or undetectable levels of *HER2* expression. An analysis of our patient cohort echoed these observations where 45% of patients expressed low *HER2* staining, demonstrating that the staining patterns in humans were consistent with our preclinical mice studies. The expression of *HER2* in our patient cohort was consistent with other published data on *HER2* expression in HNSCC tumors [6, 7, 19].

*HER2*‐targeted therapies, including antibody‐based treatments like trastuzumab and small molecule inhibitors such as Lapatinib and Afatinib, have historically shown ineffectiveness in treating HNSCC. In a phase 2 trial that combined trastuzumab with paclitaxel and cisplatin for 54 patients with recurrent or metastatic HNSCC, the addition of trastuzumab did not improve response rates [20]. Similarly, modest outcomes were observed in trials assessing trastuzumab's efficacy in salivary gland ductal carcinoma [7]. Phase 2 clinical trials involving Lapatinib, a tyrosine kinase inhibitor targeting *HER2* and *EGFR*, have also yielded disappointing results. One study showed no complete or partial responses in R/M HNSCC patients treated with Lapatinib, and another study comparing Lapatinib with placebo before chemoradiotherapy reported no significant differences in objective response rates [21, 22, 23]. These challenges underscore the significance of our preclinical findings which indicate that T‐DXd can delay tumor progression in HNSCC, especially noteworthy given the drug's breakthrough therapy designation by the FDA in August 2023 for unresectable or metastatic *HER2*‐positive solid tumors without satisfactory alternative treatments, and for *HER2*‐positive metastatic colorectal cancer patients after ≥ 2 prior regimens. Data from a phase 2 study showed objective partial responses in four patients with HNSCC who were treated with T‐DXd, highlighting the critical role it might play in the future [24]. Given its authorization for patients with breast cancer exhibiting low *HER2* expression, our evidence suggests that T‐DXd could also serve as an effective treatment for HNSCC. This marks a significant breakthrough in therapeutic strategies for HNSCC, especially considering the previous inefficacy of *HER2*‐targeted treatments in this context. Our studies also present a novel use of optically labeled antibodies to evaluate drug delivery and receptor expression in tumors before deciding on therapeutic intervention. This approach could be particularly crucial since stratifying patients accurately before initiating therapy might offer better insights. Should T‐DXd prove effective, it would join the ranks of cetuximab, pembrolizumab, and nivolumab as another molecular therapy option for treating HNSCC patients.

## 5 Conclusion

In this study, we evaluated IRDye800 conjugated to Trastuzumab in various HNSCC cell lines and xenograft models with differing levels of *HER2* expression. We observed high specificity for trastuzumab‐IRDye800 in both *in vitro* and *in vivo* settings, with significant uptake in *HER2*‐positive cells and no uptake in *HER2*‐negative cells. Furthermore, we demonstrated the effectiveness of T‐DXd in targeting HNSCC cell lines. Since a considerable amount of HNSCC patients have low *HER2* expressing tumors, T‐DXd might be a viable therapeutic option given how low *HER2* expressing HNSCC cell lines responded. Although these findings contribute to the expanding clinical evidence supporting the use of FDA‐approved monoclonal antibodies (mAbs) for fluorescence‐guided receptor expression, more human studies are needed to concretely answer if T‐DXd has a potential role in treatment down the line and if fluorescent guided receptor expression would be able to accurately stratify patients.

## Conflict of interest

The authors declare no conflict of interest.

## Author contributions

ABN contributed to conceptualization, data collection, formal analysis, and writing of original draft. LM, MBM, and AM contributed to data collection and formal analysis. TK, HT, and NM contributed to data visualization and reviewing of draft. ER and MH contributed to conceptualization, funding acquisition, and data interpretation. All authors reviewed and approved the final manuscript and take responsibility for the integrity of the data and the accuracy of the analysis.

## Peer review

The peer review history for this article is available at https://www.webofscience.com/api/gateway/wos/peer‐review/10.1002/1878‐0261.70056.

## Supporting information


**Fig. S1.** Pharmacokinetics of Trastuzumab‐IRDye800 *in vivo* and *ex vivo*.

**Table S1.** Demographic information on HNSCC cases used for *HER2* IHC staining.

## Data Availability

Data available on request from the authors.
