# Supplementary material for: Molecular imaging predicts trastuzumab‐deruxtecan (T‐DXd) response in head and neck cancer xenograft models
Source: Mol Oncol. 2025 May 28;19(11):3193–204. doi: 10.1002/1878-0261.70056 (PMC12591327; doi:10.1002/1878-0261.70056)

**Supplementary Figure 1**: Pharmacokinetics of Trastuzumab-IRDye800 in vivo and ex vivo.

A) In vivo near-infrared fluorescence imaging (NIRF) of mice bearing FaDu xenografts after Trastuzumab-IRDye800 injection over a period of 3 days. Colored circles represent regions of interest (ROI) for mean fluorescence intensity (MFI) and tumor to background ratio (TBR) measurements (Red circle = Tumor, Black circle = Background) B) Pharmacokinetics of Trastuzumab-IRDye800 in two FaDu xenograft bearing mice. A sharp increase in TBR is seen in both mice by Day 3. C) Drug uptake in livers was quantified as MFIs (± S.D.) normalized to that of muscle, n=2-3 between livers from FaDu and UMSCC-1 xenograft mice. MFI = Mean fluorescent intensity


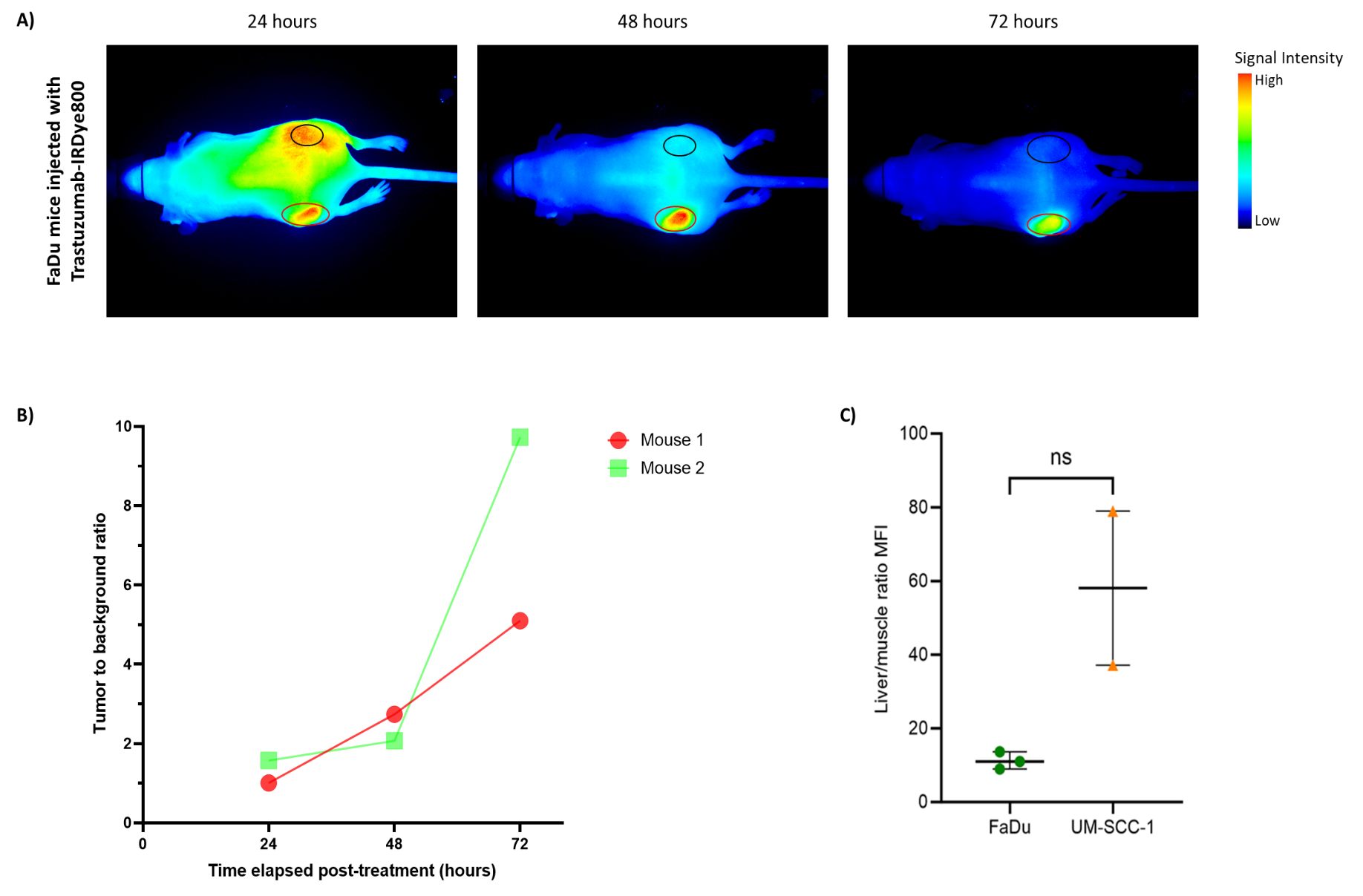

Supplement: Supplementary file 1 — Fig. S1. Pharmacokinetics of Trastuzumab‐IRDye800 in vivo and ex vivo. [file MOL2-19-3193-s001.docx]
