# Supplementary material for: Molecular imaging predicts trastuzumab‐deruxtecan (T‐DXd) response in head and neck cancer xenograft models
Source: Mol Oncol. 2025 May 28;19(11):3193–204. doi: 10.1002/1878-0261.70056 (PMC12591327; doi:10.1002/1878-0261.70056)
Supplement: Supplementary file 2 — Table S1. Demographic information on HNSCC cases used for HER2 IHC staining. [file MOL2-19-3193-s002.docx]

**Supplementary Table S1:** Demographic information on HNSCC cases.

| **Pt. ID** | **Sex** | **Race** | **Age (years)** | **Neck Dissection** | **Subsite of cancer** | **Primary Tumor Size (cm)** | **HER-2 IHC Grade** |
| --- | --- | --- | --- | --- | --- | --- | --- |
| 1 | Female | White | 29.267625 | Yes, unilateral | Tongue | 3.5 | 0 |
| 2 | Female | American | 54.554415 | Yes, unilateral | Mandible | 2.6 | 0 |
| 3 | Female | White | 70.754278 | Yes, unilateral | Tongue | 5.7 | 1+ |
| 4 | Male | White | 48.473648 | Yes, bilateral | Tongue | 6.1 | 0 |
| 5 | Female | White | 54.253251 | Yes, unilateral | Tongue | 2.2 | 1+ |
| 6 | Female | White | 59.085558 | Yes, unilateral | Tongue | 4.6 | 1+ |
| 7 | Female | White | 80.134155 | Yes, unilateral | Gingiva | 5.3 | 0 |
| 8 | Male | White | 51.838467 | Yes, bilateral | Tongue | 2.1 | 1+ |
| 9 | Male | White | 71.362081 | Yes, bilateral | Maxilla | 1.8 | 1+ |
| 10 | Female | White | 75.890486 | Yes, unilateral | Floor of mouth | 3.2 | 1+ |
| 11 | Female | White | 63.29911 | Yes, unilateral | Tongue | 2.1 | 0 |
| 12 | Male | White | 81.278576 | Yes, bilateral | Gingiva | 5.1 | 1+ |
| 13 | Female | White | 76.342231 | Yes, bilateral | Floor of mouth | 5.7 | 2+ |
| 14 | Female | White | 68.40794 | Yes, bilateral | Maxilla | 5.8 | 0 |
| 15 | Male | White | 58.398357 | Yes, bilateral | Gingiva | 2.7 | 0 |
| 16 | Male | White | 66.006845 | Yes, unilateral | Gingiva (mandibular) | 3.1 | 1+ |
| 17 | Male | White, Hispanic | 54.272416 | Yes, unilateral | Gingiva | 5.5 | 0 |
| 18 | Female | White | 51.011636 | Yes, unilateral | Gingiva | 2.2 | 0 |
| 19 | Male | Black or African American | 58.154689 | Yes, bilateral | Gingiva | 11.7 | 0 |
| 20 | Male | White | 40.752909 | Yes, bilateral | Tongue | 3.7 | 1+ |
| 21 | Female | White | 55.178645 | Yes, unilateral | Buccal | 3.6 | 0 |
| 22 | Male | White | 67.394935 | Yes, bilateral | Mandible | 2.2 | 1+ |
| 23 | Female | White | 65.519507 | No | Lip | 2.4 | 0 |
| 24 | Male | White | 74.395619 | Yes, unilateral | Mandible | 8.4 | 0 |
| 25 | Female | White | 54.102669 | Yes, unilateral | Floor of mouth | 5.8 | 0 |
| 26 | Male | White | 58.521561 | Yes, bilateral | Tongue | 5.7 | 1+ |
| 27 | Male | White | 86.083504 | Yes, unilateral | Buccal | 4.4 | 0 |
| 28 | Male | White | 80.487337 | Yes, bilateral | Mandible | 8.0 | 0 |
| 29 | Male | White | 49.486653 | Yes, unilateral | Tongue | 5.2 | 0 |
| 30 | Female | White | 93.390828 | Yes, bilateral | Gingiva | 4.6 | 0 |
| 31 | Male | White, Hispanic | 64.410678 | Yes, unilateral | Buccal | 5.4 | 0 |
| 32 | Male | White | 69.990418 | Yes, unilateral | Tongue | 2.3 | 0 |
| 33 | Female | White | 65.831622 | Yes, unilateral | Tongue | 3.6 | 1+ |
| 34 | Male | White | 53.880903 | Yes, unilateral | Mandible | 6.7 | 1+ |
| 35 | Male | White | 51.739904 | Yes, unilateral | Buccal | 3.4 | 1+ |
| 36 | Male | White | 67.485284 | Yes, unilateral | Tongue | 5 | 2+ |
| 37 | Female | White | 73.648186 | Yes, unilateral | Larynx | 2.5 | 1+ |
| 38 | Male | White | 57.804244 | Yes, bilateral | Tongue | 5.3 | 1+ |
| 39 | Male | White | 59.392197 | Yes, unilateral | Retromolar trigone | 1.5 | 1+ |
| 40 | Male | White | 81.94935 | Yes, unilateral | Gingiva | 2.8 | 1+ |
